# Spontaneous conception outcome in infertile women after four-dimensional hysterosalpingo-contrast-sonography

**DOI:** 10.1186/s12884-020-03315-x

**Published:** 2020-10-20

**Authors:** Yu Liu, Ning Zhang, Yanni He, Jiayao Shi, Meijun Zhou, Jingjiao Xu, Hongmei Liu

**Affiliations:** 1 Department of Ultrasound, Institute of Ultrasound in Musculoskeletal Sports Medicine, Guangdong Second Provincial General Hospital, Guangzhou, 510317 Guangdong PR China; 2 The Second School of Clinical Medicine, Southern Medical University, Guangzhou, Guangdong PR China

**Keywords:** Spontaneous conception, Infertility, Tubal patency, Hysterosalpingo contrast, Sonography

## Abstract

**Background:**

Four-dimensional hysterosalpingo-contrast sonography (4D-HyCoSy) is the preferred way for evaluating fallopian tubal patency and it associated with higher rate of spontaneous conception. However, Few studies have evaluated the influencing factors of spontaneous conception in 4D-HyCoSy and suggested ways to choose treatment options after 4D-HyCoSy. The study was to evaluate the correlation between spontaneous conception outcome and the patients’ clinical characteristics as well as tubal patency in infertile women to provide reference on ways to manage the patient after 4D-HyCoSy.

**Methods:**

This was a retrospective study and analysis of two hundred and eighty three (283) infertile patients who underwent a 4D-HyCoSy between December 2014 and October 2017 in our center. Eligible patients were those whose partners semen parameters were normal when based on World Health Organization (WHO) criteria, and had spontaneous conception without clinical interventions after 4D-HyCoSy.

**Result(s):**

One hundred and sixteen patients (40.9%) conceived spontaneously and the mean conception time was (8.8 ± 0.3) months. Within a year after 4D-HyCoSy, the spontaneous conception rate was highest in type VI(62.5%), followed by type IV (46.2%), type III (44.4%), type V (39.4%), type II (33.9%) and type I (14.8%). With Cox regression analysis, two factors associated with spontaneous conception outcome appeared to increase spontaneous conception rate: patients with type IV or type VI tubes and duration of infertility less than 2 years. The age, type of infertility, multiparas, history of pelvic surgery, history of uterine cavity operation, uterine fibromyomata and polycystic ovary were unrelated to spontaneous conception outcome after 4D-HyCoSy.

**Conclusion(s):**

This study showed that some infertile women could succeed in spontaneous conception after 4D-HyCoSy. Hence, We recommend the usage of 4D-HyCoSy as first line for tubal patency test and infertile patients should be advised to accept 4D-HyCoSy examination as soon as possible. Expectant treatment of about 8–9 months is reported to be feasible for infertile women whose 4D-HyCoSy findings showed one tube patency or poor patency. Alternatively, an immediate clinical intervention is recommended for those with bilateral obstructed tubes .

## Background

Fallopian tubal Factors accounts for approximately 30 to 35% of infertility among women of childbearing age [1–3]. Therefore, an accurate evaluation of tubal patency is a fundamental step during clinical management decision-making for infertile women [4–6].

Laparoscopy chromopertubation (LC) is considered as the gold standard for evaluating tubal patency. However, LC requires general anesthesia and hospitalization [7]. Hysterosalpingography (HSG) is a traditional imaging method to evaluate tubal patency, but its clinical use is limited by the risk of iodine allergy and radiation [6, 8]. Ultrasound evolved from two-dimensional contrast modes to three-dimensional and four-dimensional contrast modes [2]. Conventional two-dimensional hysterosalpingo-contrast sonography (2D-HyCoSy) has limitations in that signals from the total length of the tube and full contour of the uterine cavity have rarely been depicted in a single scanning plane because of tubal tortuosity and limited detection angles of the ultrasound beam [9, 10]. With the introduction of four-dimensional hysterosalpingo-contrast sonography (4D-HyCoSy), a diagnostic accuracy of 87.5–92.9% [2, 3, 11],the use of ultrasound in reproductive field has gradually increased. As opposed to 2D-HyCoSy and three-dimensional hysterosalpingo-contrast sonography (3D-HyCoSy), 4D-HyCoSy enables observation of the entire course of fallopian tube contrast development rather than an instant capture [2, 12–14].

Our clinical follow-up data and previous studies showed that some infertile women succeed in spontaneous conception after 4D-HyCoSy. Thus, expectant treatment is recommended to reduce overtreatment and save medical resources due to the therapeutic effects of tubal flushing test [15]. However, Few studies have suggested ways to choose treatment options. i.e., expectant treatment or clinical interventions based on the primary clinical characteristics of infertile women and their evaluation results of 4D-HyCoSy on tubal patency. In addition, few studies have suggested the expectant treatment time after 4D-HyCoSy.

The study aimed to analyze the correlation between the spontaneous conception outcomes of infertile women after 4D-HyCoSy and their underlying clinical conditions as well as the tubal patency, to provide reference on ways to manage the patient after 4D-HyCoSy, as well as carry out further clinical interventions.

## Methods

### Study area and design

This retrospective study was approved by the Ethics Committee of Guangdong Second Provincial General Hospital (2018-CSkWZ-009). All participants had signed informed consent before examinations.

### Patients selection

**Fig. 1:**
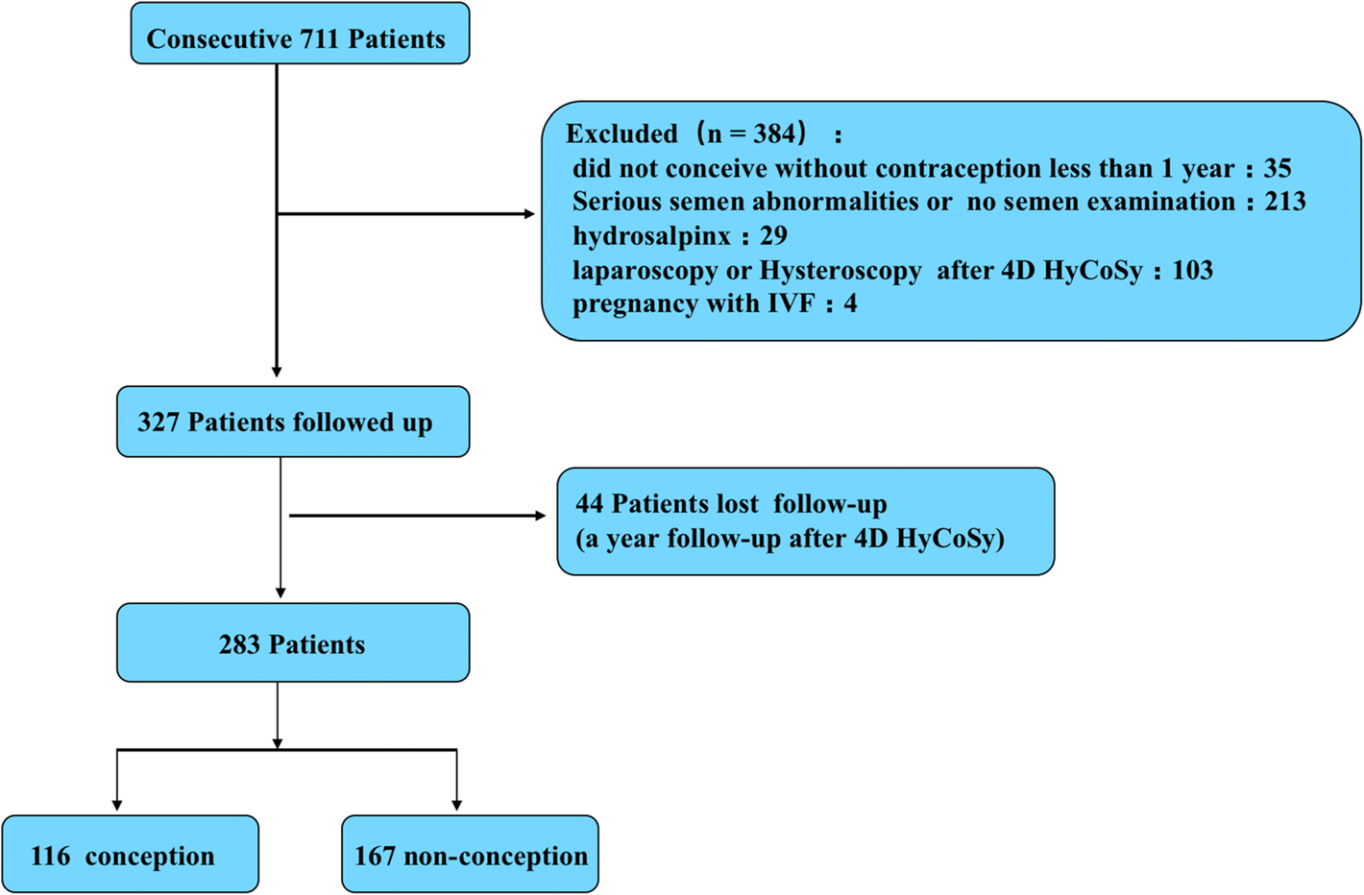
The flowchart of the study

All the records of the seven hundred and eleven (711) patients who underwent the 4D-HyCoSy in our center between December 2014 and October 2017 were extracted for in depth analysis. Basic clinical data extracted from the records included age, duration of infertility, type of infertility, multipara, previous pelvic inflammatory disease, previous pelvic surgery, previous intrauterine surgery and abortions. In order to determine the spontaneous conception of infertile women after 4D-HyCoSy, it was necessary to select patients who conformed to two major criteria: 1. the patient’s husband showed no evident cause for infertility [16]. 2. No clinical interventions (IVF or hysteroscopy and laparoscopic surgery) were given after 4D-HyCoSy. To conform to these criteria, the exclusion criteria were as follows: women did not conceive without contraception less than a year (35 patients), semen abnormalities (according to WHO 2010 guideline) or no semen examination (213 patients), hydrosalpinx by ultrasound examination (29 patients), hysteroscopy and laparoscopic surgery (103 patients) or in vitro fertilization (IVF) (4 patients) within a year after 4D-HyCoSy, According to the exclusion criteria, 327 patients were enrolled in our follow-up study (Fig. 1).

### 4D-HyCoSy

A Voluson E8 Expert (GE Healthcare, Zipf, Austria) With RIC5–9-D volume probe (5–9 MHz) was used. Equipment settings for 4 dimensional contrast sonography were as follows: volume box angle at 179°, volume angle at 120°, quality at low, direction at up/down, render mode at gradient light and threshold, Low at < 20, adjusted according to the image [14, 17]. The contrast agent was prepared by adding 5 ml of 0.9% sterile saline solution to 59 mg of SF6 Sonovue freeze-dried powder (Bracco International BV, Amsterdam, the Netherlands), then 2.5 ml SonoVue solution was aspirated and diluted it into a 15 ml suspension with the 0.9% of saline solution.

The 4D-HyCoSy was performed within 3 to 10 days after menstruation. Each examination was performed by an experienced physician with more than 5 years of ultrasonographic diagnosis. The patient was in lithotomy position with her vulva and cervix routinely disinfected with an iodinated solution (Effective iodine content: 0.45–0.55%, Shanghai Likang Disinfection Technology Co., Ltd). The condition of the uterus, ovaries and pelvic cavity was evaluated by 2-dimensional transvaginal sonography and the results were recorded (uterine fibromyoma, uterine cavity lesions such as endometrial polyps or adhesions and polycystic ovaries). Next the 4D-HyCoSy was performed. An appropriate initial plane was selected by positioning the vaginal probe at the level of the sectional plane of the uterus, with slight adjustment to allow visualization of bilateral uterine horns and surrounding tissues. 4D-HyCoSy was activated while keeping the probe at the same position with the region of interest as wide as possible. Fifteen millimeters (15 ml) of the contrast agent earlier prepared was then insufflated and adjusted in accordance with the development in the fallopian tube(s) such as spillage at the fimbriae of the contrast in the pelvis. The contrast agent was passed through the catheter into the uterine cavity, following which the flow of the contrast agent in the fallopian tube and the overflow at the tubal fimbriae were observed. The dynamic enhanced volume images were saved and analyzed after the injection of the contrast medium [2, 11, 14].

### Criteria for tubal patency [14] (Fig. 2)

#### Fallopian tube patency

The contrast agent filled the whole uterine cavity that quickly flowed into the fallopian tube and sprayed at the fimbria of the tube. In addition, the passage of the tube was soft and naturally directed downward.

**Fig. 2:**
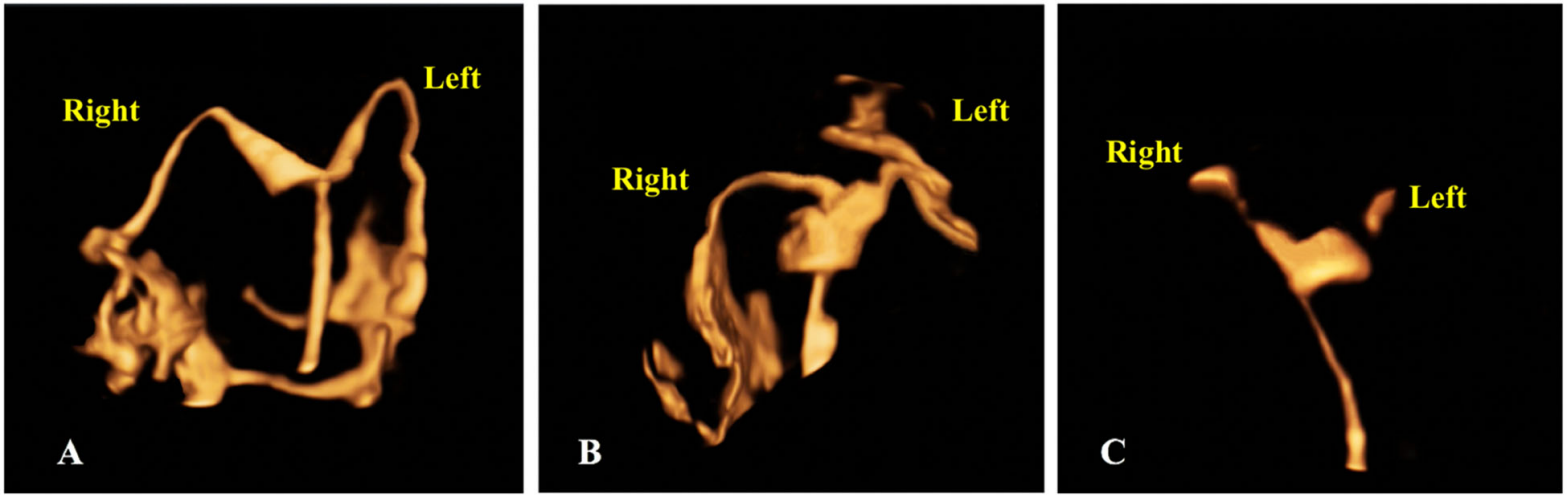
Different types of fallopian tubes on 4D-HyCoSy. **a**. The tubes were patent, and the passage of the tube was soft and naturally. **b**. The left tube was poor patency, and the entire fallopian tube was stiff, angled, circuitous and directed upward. **c**. Both fallopian tubes were obstructed, we could not see the entire passage of the tubes or the spillage at the fimbria of the tube

#### Poor patency of fallopian tube

The entire fallopian tube and the spillage at the fimbria of the tube were visible, but the passage of the tube was stiff, discontinuous, filamented, angled, circuitous, and directed upward.

#### Fallopian tube obstruction

Resistance built-up following the injection of the contrast agent. We could not see the entire passage of the tube or the spillage at the fimbria.

### Classification of fallopian tube

According to the bilateral tube patency, patients were divided into six categories: type I, defined as bilateral fallopian tubes obstruction; type II, defined as one obstructed fallopian tube with poor patency in the other; type III, defined as one obstructed fallopian tube with patent in the other; type IV, defined as bilateral fallopian tube poor patency; type V, defined as one fallopian tube poor patency with patent in the other; type VI, defined as bilateral fallopian tubes patent.

### Outcomes

The outcome of spontaneous conception defined as conception by sexual intercourse and a sonographically visible fetal sac within the uterine cavity. Patients were followed up once a month by telephone, clinical, ultrasound examinations after 4D-HyCoSy. The follow-up lasted for 12 months or till the patient succeeded in the first spontaneous conception.

### Statistical analysis

All statistical analyses were conducted with SPSS version 23.0 software for windows (SPSS Inc., Chicago, IL, USA). The data were expressed as $\overline{x}$ ± SD or percentage. The chi-square test was used to compare the difference among groups for counting data. Kaplan–Meier method was performed to calculate cumulative conception rates and conception time after 4D-HyCoSy. Log-rank test was performed to evaluate the clinical and ultrasound factors on spontaneous conception that included age, duration of infertility, infertility type, previous pelvic surgery, history of abortion, history of intrauterine surgery, history of pelvic inflammatory disease, multiparas or not, uterine fibromyoma, lesion of uterine cavity (adhesions or/and polyps), polycystic ovary and tubal patency. Next, a stepwise Cox regression was performed to analyze the potential confounding affecting factors associated with spontaneous conception, and a *P* value < 0.05 was considered significant.

## Results

### Basic characteristics of the patient

A total of seven hundred and eleven (711) patients performed 4D-HyCoSy in this study, and 327 (327/711,46.0%) met the inclusion criteria, Each case was followed up for a year. A total of 44 patients (44/327,13.5%) were lost follow-up, and 283 patients were finally included in our study (Fig. 1). six patients undergo repeated procedure due to severe venous intravasation. No Patients had a severe vasovagal reaction requiring resuscitation. The age of the participants ranged from 20 to 46 years, with an average age of (30.4 ± 5.4) years. The duration of infertility among patients ranged from 1 to 10 years, with a mean of (2.0 ± 1.7) years.

### Natural conception outcome

One hundred sixteen (116) infertile women succeeded in spontaneous conception in a year after 4D-HyCoSy and they were carried to term. Also, an ectopic conception occurred. The cumulative conception rate was 40.9% and the mean conception time was (8.8 ± 0.3) months. The conception rate of 1–3 months,1–6 months,1-9 months and 1–12 months was 21.5%, 30.4%, 38.2%, 40.9%, respectively; and the conception rates of 1–3 months, 4–6 months, 7–9 months and 10–12 months accounted for 52.6, 20.7, 19.8 and 6.9% of the total conception rates, respectively.

### Factors on spontaneous conception

The three factors (tubal patency, duration of infertility, uterine cavity lesions) selected by univariate analysis were included in multivariate regression analysis. The results showed that spontaneous conception outcome after 4D-HyCoSy was the result of multiple factors, i.e. the spontaneous conception rates increased with decreasing infertility duration and good tubal patency (Table 2). No significant difference was observed in the age, type of infertility, multiparas, history of pelvic surgery, history of pelvic inflammation, abortion, uterine cavity operation, uterine fibromyomata, and polycystic ovary between conception group and non-conception group after 4D-HyCoSy (*P* > 0.05) (Table 1).

**Table 1:** Basic characteristics between conception group and non-conception group

Characteristic	Conception (***n*** = 116)	Non-conception (***n*** = 167)	***P***
**Age group (*n*** **/%)**			0.155
<30 years	69 (46.3)	80 (53.7)	
30–35 years	28 (35.9)	50 (64.1)	
≧35 years	19 (33.9)	37 (66.1)	
**Infertility type (n/%)**			0.365
Primary infertility	52 (32.8)	84 (61.8)	
Secondary infertility	64 (43.5)	83 (56.5)	
**Duration of infertility** **group (n/%)**			0.000
<2 years	83 (52.5) ^a, b^	75 (47.5) ^a, b^	
2-3 years	20 (29.4)	48 (70.6)	
≧3 years	13 (22.8)	44 (77.2)	
**History of pelvic** **Surgery (n/%)**			0.505
yes	18 (36.7)	31 (63.3)	
no	98 (41.9)	136 (58.1)	
**History of abortion (n/%)**			0.420
yes	40 (44.4)	50 (55.6)	
no	76 (39.4)	117 (60.6)	
**History of uterine cavity operation (n/%)**			0.531
yes	45 (38.8)	71 (61.2)	
no	71 (42.5)	96 (57.5)	
**Multiparas (n/%)**			0.726
yes	37 (42.5)	50 (57.5)	
no	79 (40.3)	117 (59.7)	
**History of pelvic inflammation (n/%)**			0.064
Yes	10 (27.0)	27 (73.0)	
No	106 (43.1)	140 (56.9)	
**Polycystic ovary (n/%)**			0.747
Yes	19 (41.3)	27 (58.7)	
No	104 (43.9)	133 (56.1)	
**lesion of the uterine cavity (n/%)**			0.042
yes	13 (27.7)	34 (72.3)	
no	103 (43.6)	133 (56.4)	
**Fibromyomata (n/%)**			0.584
yes	16 (37.2)	27 (62.8)	
no	100 (41.7)	140 (58.3)	

^a^Difference in duration of infertility group between **<** 2 years and ≧3 years

^b^Difference in duration of infertility group between**<** 2 years and 2 to 3 years

**Fig. 3:**
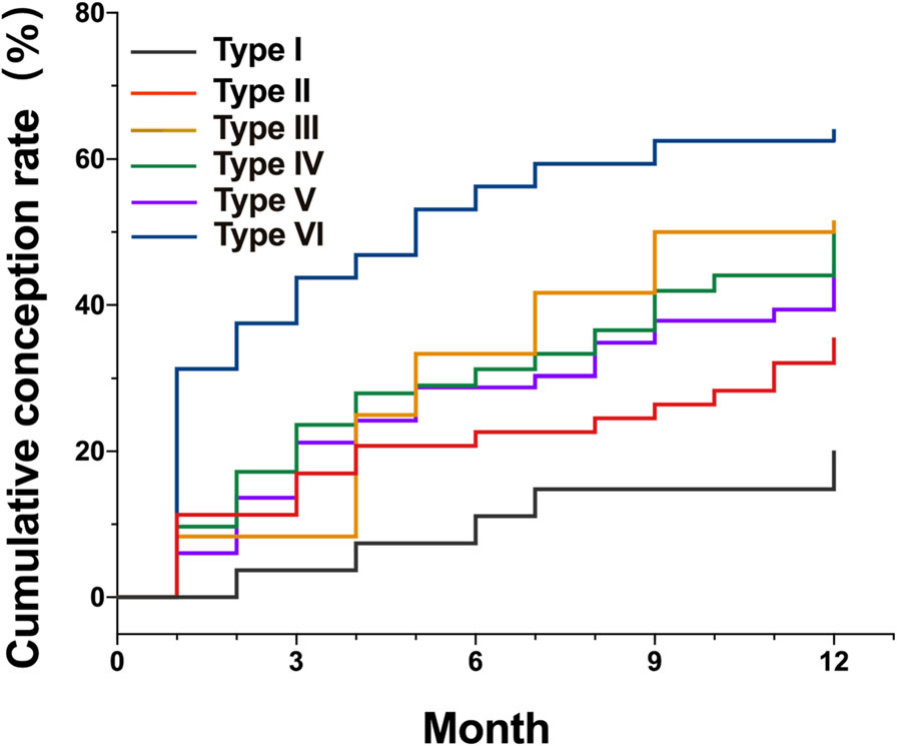
Total cumulative conception rate in different fallopian tubes patency. The conception rate was highest in type VI (62.5%), followed by type IV (46.2%), type III (44.4%), type V (39.4%), type II (33.9%) and type I (14.8%) (*P* < 0.01)

**Fig. 4:**
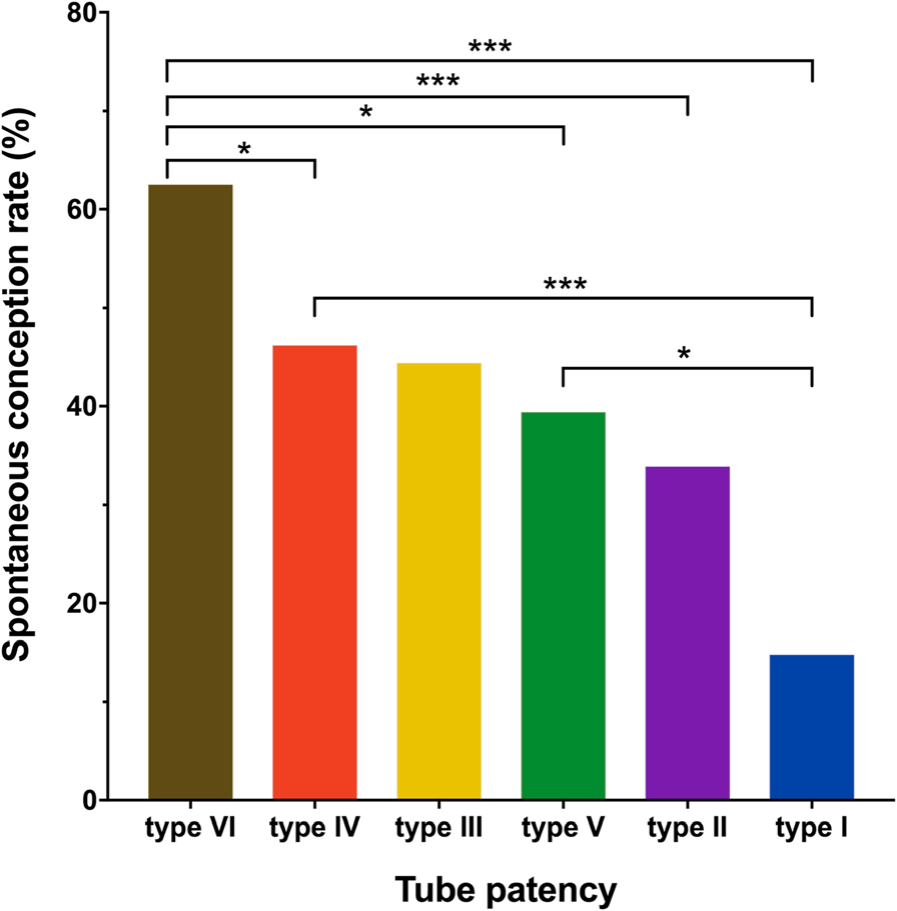
The comparison of conception rate among groups with different types of fallopian tubes. *: *p*<0.05; **: *p*<0.01; ***: *p*<0.001

4D-HyCoSy results suggested that there were 27 cases of type I, 56 cases of type II, 9 cases of type III, 93 cases of type IV, 66 cases of type V, and 32 cases of type VI, respectively. Within a year after 4D-HyCoSy, the spontaneous conception rate was highest in type VI, reaching 62.5%, followed by type IV (46.2%), type III (44.4%), type V (39.4%), type II (33.9%) and type I (14.8%) (*P* < 0.01) (Figs. 3 and 4). Further comparison between the two groups (Due to the small number of type III, no statistical analysis was performed) showed that the conception rate of type VI significantly exceeded that of type IV and type V. There was no significant difference in conception rate between type IV and type V. Meanwhile, the conception rate of type VI, type IV and type V was significantly higher than that of type I (Fig. 4). Cox regression analysis showed fallopian tube patency was significantly associated with spontaneous conception, and the conception rate of infertile women with type IV and type VI increased significantly after 4D-HyCoSy.The mean conception time was (5.0 ± 2.1) months in type VI, (8.6 ± 0.5) months in type V and (9.0 ± 0.5) months in type IV, respectively.

**Fig. 5:**
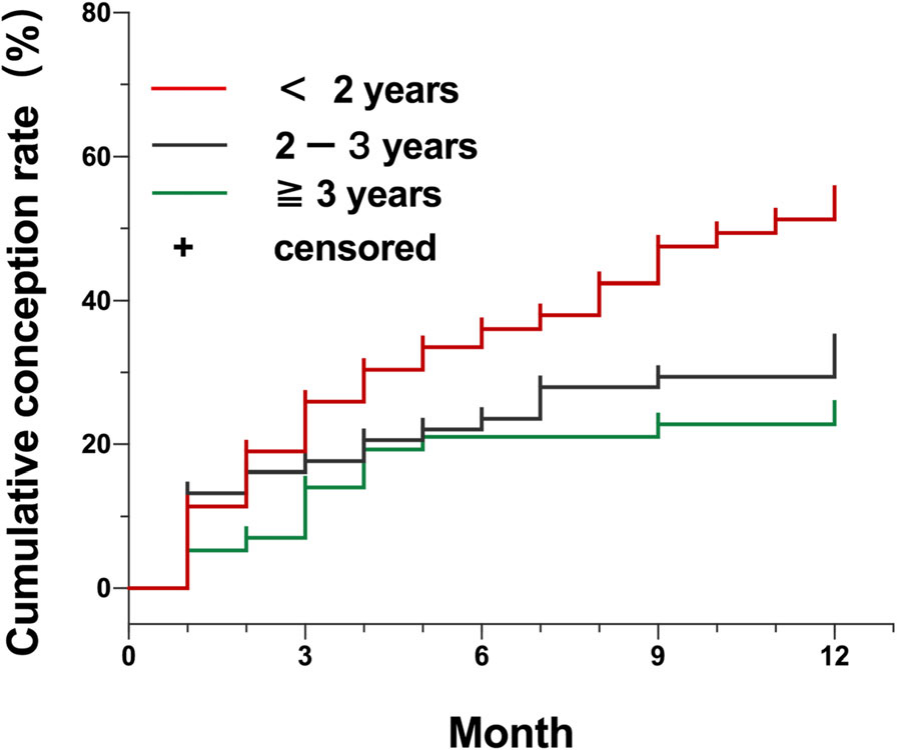
Total cumulative conception rate in different sterility duration. The conception rate was highest in duration of infertility less than 2 years(52.5%), followed by duration of infertility ranging 2 to 3 years (29.4%) and the duration of infertility more than or equal to 3 years (22.8%)

The conception rate was highest among women who were infertile for less than 2 years (52.5%), followed by duration of infertility ranging 2 to 3 years (29.4%) and the duration of infertility more than or equal to 3 years (22.8%)(*P* < 0.05) (Table 1 and Fig. 5). Cox regression analysis showed that duration of infertility was significantly associated with spontaneous conception (Table 2). The conception rate of infertile women with duration of infertility less than 2 years increased significantly after 4D-HyCoSy (Table 2). The mean conception time was (8.2 ± 0.4) months in patients with duration of infertility less than 2 years.

**Table 2:** The influencing factors of spontaneous conception after 4D-HyCoSy

Factors	Kaplan-Meier analysis		Cox regression	
	HR(95%CI)	***P***	HR(95%CI)	***P***
**Tubal patency**				
Type VI	6.87 (2.35, 20.11)	0.000	6.04 (2.06, 17.75)	0.001
Type IV	3.8 (1.37, 10.59)	0.011	3.22 (1.15, 9.01)	0.026
Type III	3.77 (1.01, 14.02)	0.048	3.48 (0.93, 13.02)	0.064
Type V	3.12 (1.09, 8.95)	0.034	2.59 (0.90, 7.46)	0.077
Type II	2.51 (0.85, 7.42)	0.095	2.30 (0.78, 6.83)	0.132
**Duration of infertility group**				
<2 years	2.71 (1.51, 4.87)	0.001	2.76 (1.53, 4.97)	0.001
2-3 years	1.37 (0.68, 2.76)	0.375	1.46 (0.72, 2.94)	0.293
**Disease of the uterine cavity**				
no	1.74 (0.98, 3.09)	0.061	1.69 (0.94, 3.03)	0.079

*HR* Hazard ratio, *CI* Confidential interval

Tubal patency group was compared with type I group. Duration of infertility group was compared with duration of infertility more than or equal to 3 years. Disease of the uterine cavity group was compared with those without. Type I, defined as both fallopian tubes obstructed, Type II, defined as one fallopian tube obstructed with poor patency in the other; type III, defined as one fallopian tube obstructed with patent in the other; type IV, defined as both fallopian tubes poor patency; type V, defined as one fallopian tube poor patency with patent in the other; type VI, defined as both fallopian tubes patent

The conception rate was significantly low in women with lesion of uterine cavity (endometrial polyp or adhesion of uterine cavity) (Table 1).

## Discussion

Our study demonstrated that some infertile women could succeed in spontaneous conception after 4D-HyCoSy and their conception rates could be up to 40.9%. The mean conception time was (8.8 ± 0.3) months within a year after 4D-HyCoSy. This may be an indication that infertile women could have expectant treatment for a certain time to reduce overtreatment and clinical risks. The positive effect of 4D-HyCoSy is probably due to mechanical action of the contrast agent. The passage of liquid breaks up minor adhesion within the tubes [4, 15].

The conception rate of infertile women within a year after 4D-HyCoSy was higher than those reported by Chunyan Gao et al. (40.9% vs. 26.59%) [15]. There may be two reasons. Firstly, this may due to the fact that we excluded some infertile patients with semen abnormalities of their husbands. Secondly, it is the relatively large proportion of bilateral tube patency (67.5% vs. 62.0%) and small proportion of bilateral tube obstruction (9.5% vs. 13.2%) in our study. In addition, the incidence of spontaneous conception in a year after 4D-HyCoSy in our study was higher than those who accepted hysterosalpingo-foam sonography (HyFoSy) used ExEm-gel (40.9% vs. 19%) [18]. On the other hand, the incidence of conception rate in a year after tubal flushing among those who accepted HSG used oil contrast (32.1%) or water contrast (23.6%) was relatively lower than our study (40.9%) [19]. Our results showed that the mean conception time was (8.8 ± 0.3) months within a year after 4D-HyCoSy, which indicated that some infertile women could succeed in spontaneous conception within a short time after 4D-HyCoSy. Furthermore, we found out that fallopian tubes patency was significantly associated with spontaneous conception, which is consistent with the previous studies [4, 15]. These studies suggested that infertile women with type VI should be considered at least 5 months for expectant treatment, whereas 9 months is typical for type IV and type V infertile women.

In our study, there were only 9 women with type III tubes, and their conception rate reached 44.4%. In this group, women with infertility who succeed in achieving spontaneous conception had short duration of infertility, mostly about 1 year. Therefore, for infertile women with one tube obstruction but short duration of infertility, 8 to 9 months of expectant treatment should be considered.

Theoretically, it was challenging to achieve spontaneous conception among type I infertile patients, and therefore, clinical interventions was necessary [20]. Our study included exceptional cases, i.e. Four infertile patients achieved spontaneous conception, probably due to the passage of liquid through the tube that removed the buildup of debris inside it, and the other might be due to the false-positive results of tubal spasm during 4D-HyCoSy.

Regression analysis showed that short duration of infertility (less than 2 years) was significantly associated with spontaneous conception after 4D-HyCoSy, which was consistent with published studies [4]. The reason might be that long-term tubal obstruction caused by chronic inflammatory reaction damaged the internal structures including the cilia, and made it difficult to achieve spontaneous conception [21]. Therefore, we strongly recommend that infertile women should undergo 4D-HyCoSy examination as early as possible. Alternatively, infertile women with duration of infertility less than 2 years after 4D-HyCoSy can consider about 8 to 9 months of expectant treatment.

However, our study has two significant limitations. We did not compare the spontaneous conception rate of infertile women who did not undergo 4D-HyCoSy in outpatient clinics or underwent immediate clinical management (laparoscopic surgery, assisted reproduction treatment) after 4D-HyCoSy, which need further research in future.

The clinical importance of our results is that some infertile patients could undergo expectant treatment for a certain time after 4D-HyCoSy, which could reduce overtreatment and clinical risks and save medical resources.

## Conclusions

This study showed that some infertile women could achieve spontaneous conception after 4D-HyCoSy. Hence, We recommend the usage of 4D-HyCoSy as first line for tubal patency test and infertile patients should be advised to accept 4D-HyCoSy examination as soon as possible. Expectant treatment of about 8–9 months is reported to be feasible for infertile women whose 4D-HyCoSy findings showed one tube patency or poor patency. Alternatively, an immediate clinical intervention is recommended for those with bilateral obstructed tubes .

## Data Availability

The data used and/or analysed during the current study available from the corresponding author on reasonable request.

## Abbreviations

4D-HyCoSy: Four-dimensional hysterosalpingo-contrast sonography
WHO: World Health Organization
LC: Laparoscopy chromopertubation
HSG: Hysterosalpingography
HyFoSy: Hysterosalpingo-foam sonography
2D-HyCoSy: Two-dimensional hysterosalpingo-contrast sonography
3D-HyCoSy: Three-dimensional hysterosalpingo-contrast sonography
IVF: In vitro fertilization
